# The rationale of using angiotensin receptor blocker instead of pulmonary vasodilators to treat pulmonary hypertension in bronchopulmonary dysplasia: a case report and literature review

**DOI:** 10.3389/fped.2025.1504180

**Published:** 2025-05-19

**Authors:** Lars Lindberg

**Affiliations:** Institution of Clinical Sciences, PICU, Children’s Hospital in Lund, Skane University Hospital, Lund University, Lund, Sweden

**Keywords:** bronchopulmonary dysplasia, pulmonary hypertension, angiotensin receptor blocker, phosphodiesterase type 5 inhibitor, prematurity

## Abstract

This case report highlights the challenges in treating bronchopulmonary dysplasia (BPD) in a premature infant with severe pulmonary hypertension, recurrent pulmonary hypertensive crises, and the need of 100% oxygen to achieve acceptable arterial oxygen saturations. Key factors in the infant's improvement involved switching from pulmonary vasodilation to systemic afterload reduction using losartan, an angiotensin II type 1 receptor blocker. This alteration in treatment strategy led to a pronounced and prompt decrease in pulmonary arterial pressure, reduced oxygen dependency and resolution of pulmonary hypertensive crises. The infant's remarkable clinical response suggests that the pulmonary hypertension in BPD may have a pulmonary post-capillary cause, possibly driven by angiotensin II. A literature review corroborates this revision of the current understanding of the pathophysiologic mechanism involved in BPD and suggests that therapies targeting the renin-angiotensin-aldosterone system rather than pulmonary vasodilation may be an effective treatment strategy.

## 1 Introduction

Bronchopulmonary dysplasia (BPD) is a chronic neonatal lung disease commonly seen in extremely premature infants and along with increasing survival rates a growing burden in neonatal intensive care units (NICU) (1–3). BPD is characterized by injury to the lung parenchyma and vascular compartments (4–6) with histological evidence of alveolar destruction, epithelial injury, smooth muscle hyperplasia, diffuse fibrosis, inflammatory cell infiltration, and pulmonary venous congestion (7–11). Commonly used therapeutic measures in the NICU, such as long term oxygen supplementation and mechanical ventilator support, can themselves exacerbate lung damage via activation of inflammation and oxidative stress pathways (12, 13).

A frequent complication of BPD is the development of pulmonary hypertension (PH) (14–18). PH in BPD is most often interpreted as having a pre-capillary origin, and thus treated with pulmonary vasodilators, such as phosphodiesterase inhibitors, inhaled nitric oxide (iNO), inhaled iloprost, and endothelin receptor antagonists (15, 19). These drugs can temporarily improve oxygenation and reduce PH, but pulmonary vasodilators neither prevent the development of BPD nor lower mortality rates (20–27). This indicates that PH in severe BPD is associated with an increase in morbidity and mortality (18), but not the cause and accordingly a potential confounder. The findings by Cohen et al. (28) that a cohort with BPD and PH treated with sildenafil had the highest overall mortality (26%) of all their cohorts compare with the findings by Yung et al. (29) that a limited use of sildenafil had an extraordinarily low mortality rate of only 5% in their children with severe BPD and PH, indirectly indicate that the use of pulmonary vasodilators may not be appropriate. The understanding of PH in BPD as a pre-capillary phenomenon that should be treated with pulmonary vasodilators warrants a reconsideration.

Moreover, research indicates that left sided cardiac strain caused by systemic hypertension, likely due to increased systemic vascular resistance, is common in cohorts of children with BPD (30–32). This contributes to a subtle left ventricular diastolic dysfunction, increased left atrial pressure, pulmonary venous congestion and ultimately the development of an insidious pulmonary post-capillary PH (24, 33). Elevated left atrial pressure is associated with higher mortality rate in children with BPD (33). In addition, the effectiveness to reduce PH by systemic afterload reduction via renin-angiotensin-aldosteron-system (RAAS) inhibition has been documented and supports the assumption that PH in BPD has a post-capillary rather than pre-capillary origin (34).

The presented case reports a child with severe BPD and PH, who showed a remarkable improvement after switching the treatment strategy from pulmonary vasodilators to the angiotensin receptor blocker (ARB), losartan. The pathophysiological mechanisms and rationale behind using RAAS inhbition instead of pulmonary vasodilators in BPD are discussed.

## 2 Case description

**Figure 1:**
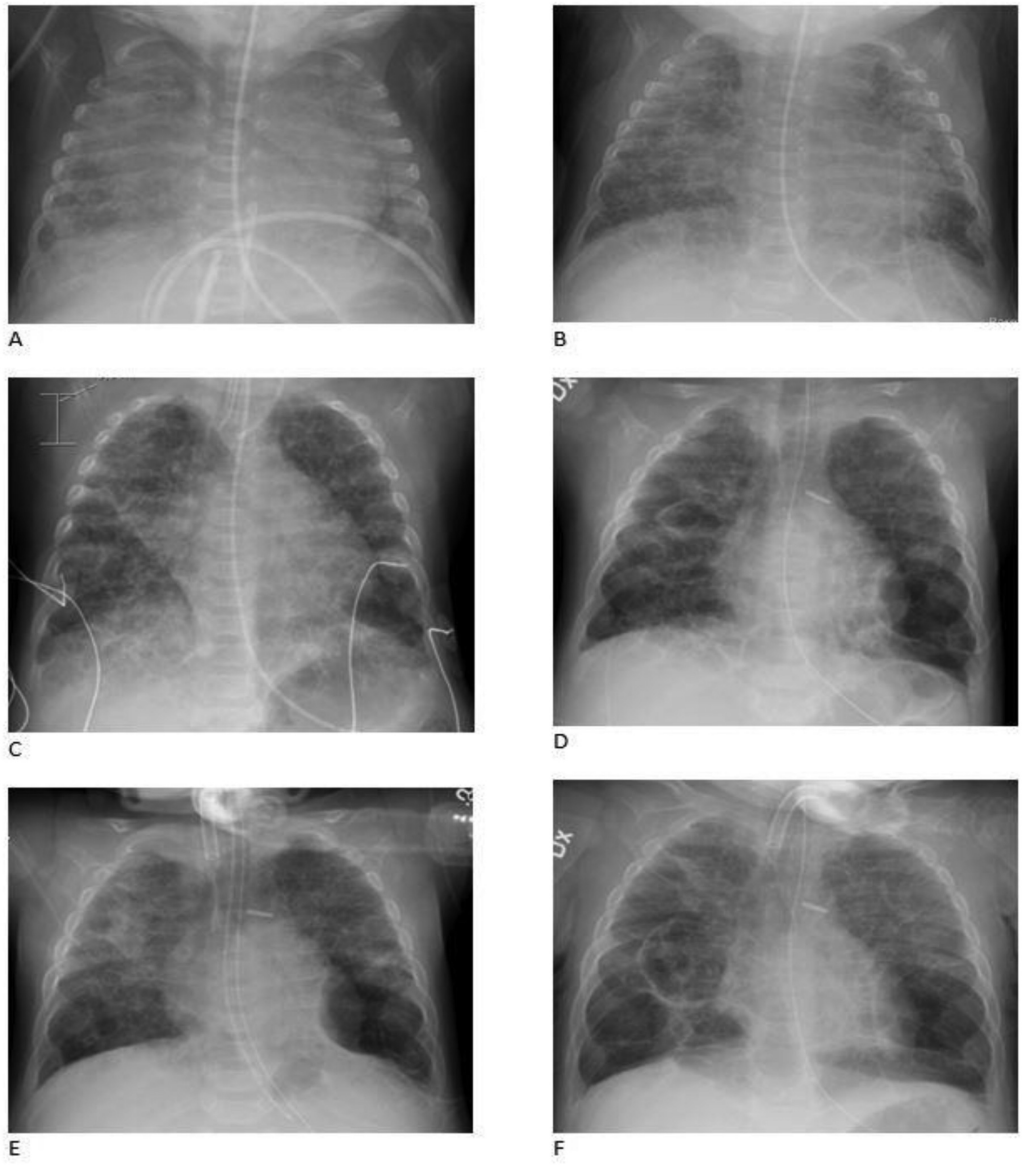
Representative chest radiographs. **(A)** PMA (post-menstrual age) 26w, **(B)** PMA 35w, **(C)** PMA 41w (before closure of the shunts), **(D)** PMA 50w (before tracheostomy), **(E)** PMA 55w (after tracheostomy) and **(F)** PMA 56w. The child developed early bilateral fine granular opacities in the pulmonary parenchyma and air bronchograms as seen in respiratory distress syndrome (RDS). Pulmonary edema could not be excluded. The granular opacities remained and became coarse. Hyperinflation and cystic changes developed and the cystic structure in left lower lobe was defined as pneumatocele. Transient apical atelectasis and non-specific parenchymal opacities occurred mainly on the right side.

This case presents a female infant born vaginally at a gestational age of 25 + 1 weeks, with a birth weight of 820 g [appropriate for gestational age (AGA)], which speaks against intrauterine growth restriction (35). She received “less invasive surfactant administration” (LISA) with Curosurf® 240 mg at 3 hours and 120 mg at 2 days of age. The infant developed severe respiratory distress syndrome (RDS) and required endotracheal intubation at 5 days of age due to hypercapnia, hypoxia and fatigue (Figure 1). Duration of invasive mechanical ventilation and non-invasive ventilation are shown in Table 1. An intraventricular hemorrhage (IVH grade 1—subependymal bleeding) was diagnosed on the right side at 3 days of age. Several follow-ups during the course of the disease did not detect any new bleedings or sign of perventricular leukomalacia (PVL).

**Table 1:** A clinical timeline.

PMA	25	26	27	28	29	30	31	32	33	34	35	36	37	38	39	40	41	42	43	44	45	46	47	48	49	50	51	52	53	54	55	56
Invasiv Vent		x	x	x	x	x										x	x	x	x								x	x	x	x	x	x
NIV	x						x	x	x	x	x	x	x	x	x					x	x	x	x	x	x	x						
∼FIO_2_	0.4	0.4–0.6	0.4–0.8	0.7–0.9	0.3	0.3	0.4	0.5	0.5	0.4	0.6	0.6	0.7	0.6	0.7–0.95	0.4	0.8–1.0	0.5–0.9	0.5	0.3–0.7	0.5 5–10l	0.5 5–10l	0.4 cs	0.5–0.7 cs	0.5–0.6 cs	0.5–1.0 cs	0.7–1.0 cs	0.6–1.0	0.8	0.6	0.4	0.4
Steroids, i.v./po	x a		x b	x c	x	x		x d	x	x				x e	x	x f	x g	x h	x i	x j	x	x	x	x	x	x	x k	x l	xm	x	x n	x
Inh.steroids								x o	x	x	x	x	x	x	x	x	x	x	x	x	x	x	x	x	x	x	x	x	x	x	x	x
PVD																		S p	S q	S	s	S	S	S	S	S	S	S,I,iNO: r s				
ARB																													L	L	L	L
Inotrope																	Mil															
AVD																	Nip															
Cultures	MRSA t		Staf u																		Rhino v								Kleb x			
Weight (g)	820				1,130							2,565									4,068						4,890					
																	Sur										Tra					

PMA, postmenstrual age in weeks [gestational age plus postnatal age (PMA = GA + PNA)]; Invasiv ventilation–mechanical ventilation with the child intubated or tracheostomized, included HFOV (high frequency oscillatory ventilation (12 days); NIV (non-invasive ventilation) was most often combined with neurally-adjusted ventilatory assist (NAVA) with either nasal sprongs or full-face mask. NIV also included periods with controlled positive airway pressure (CPAP) ventilation and bi-level positive airway pressure (BIPAP) with Trilogy or Astral ventilators; FIO2 (fraction of inspired oxygen), cs, occurrence of cyanotic spells; Steroids i.v. and p.o. were administered as betametason or hydrokortison; Inhaled steroid was administrated as budesonide; PVD, pulmonary vasodilating drugs (Sildenafil (S), inhaled iloprost (I), inhaled nitric oxide (iNO); ARB, angiotensin receptor blocker [Losartan (L)]; Mil, milrinon, only perioperative use; AVD, (arterial vasodilating drugs); Nip, (nitroprusside); Cultures, MRSA, (methicillin-resistant staphylococcus aureus); Kleb, (klebsiella pneumoniae); Rhino, (rhinovirus). Weight in gram (g); Sur, surgical closure of ASD and PDA; Tra, tracheostomy. Days of treatment and doses: Steroids: a: Day (D)1–7 Hydrocortisone (H), 0.4 mg × 2; b: D14–D20 Betametasone (B) 0.05 mg × 1–2, c: D21–D38 0.05–0.025 mg × 2; d: D52–D60 H 0.6 mg × 3–2; e: D87–D90 B 0.3 mg × 2; f: D103–D112 B 0.3 mg × 2; g: D113–D116, peri-operative prophylaxis H 7 mg × 4; h: D117–D123 B 0.25 mg × 2; i: D124–D130 0.2 mg × 2;j: D130–D184 B 0.05 mg × 1; k: D185–D187, peri-operative prophylaxis H 7 mg × 4; l: D188–D200 B 0.05–0.02 mg × 1; m: D201–D215 H 4 mg × 4; n: D215–D227 B 0.05 mg × 1. Inhaled budesonide: o: D48–D227 0.25 mg × 2; PVD: p: Sildenafil D117–D122 1 mg–2.5 mg × 3; q: D123–D201 2.7–3 mg × 3; r: iNO D199–201 10–20 ppm; s: Inhaled iloprost D199–D199 2 µg×8; Cultures: t: MRSA in throat and perineum D3, D51, D62, D110, D142, D181; u: Staphyloccoccus epidermidis in blood D20 and D199 (regarded as skin contamination, on treatment with adequate antibiotics; v: D142 Rhino in throat; x: D201 Kleb in tracheal cannula.

**Figure 2:**
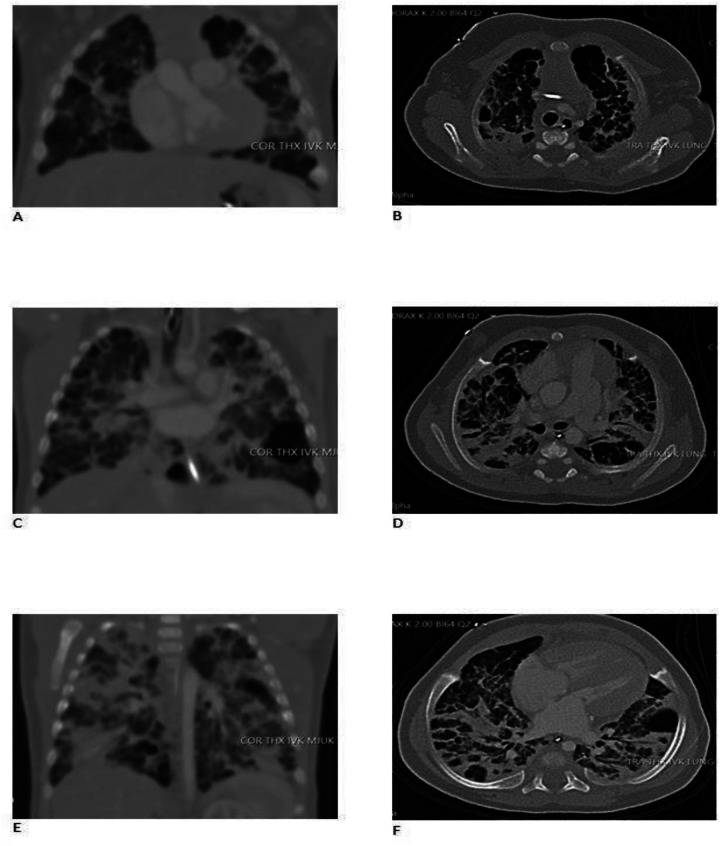
Ct scans with contrast of the chest. **(A)** CT (computed tomography) scan was performed at PMA (post-menstrual age) 41w before closure of the shunts. Frontal view on left, anterior to dorsal **(A,C,E)** and transverse view on right, apical to basal **(B,D,F)**. Pulmonary venous obstruction was excluded and the PDA and ASD could be verified. Pronounced bilateral lung parenchymal abnormalities with crazy paving, ground glass, intralobular lines, thickening of interlobular septa, and dorsal consolidation were noticed in keeping with BPD. Consolidation of the middle lobe with no suspicion of intralobular sequestration was seen. Ventrobasal in the left lower lobe an 18 × 20 × 16 mm air filled cystic structure, probable a pneumatocele was diagnosed. Several small cystic changes were seen bilateral, especially in basal parts of the lung. A mild pulmonary edema could not be excluded.

The first cardiac ultrasound was performed at 6 days of age and revealed an atrial septal defect (ASD) and a patent ductus arteriosus (PDA, Vmax 1,8 m/s), both with significant left-to-right shunting. A reversible ante-to-retrograde blood flow was noticed in mesenteric blood vessels, which indicated a significant run-off phenomenon of blood flow from the gut into the lung with diastolic left-to-right shunt in the PDA, but no NEC developed. Ibuprofen (PEDEA®) was administered intravenously with five doses with no effect on the PDA. The right ventricle was dilated due to pulmonary overcirculation and on some cardiac ultrasounds judge to be slightly hypertrophied. Left ventricle had normal systolic contractility. A CT scan ( Figure 2 ) and cardiac ultrasounds clearly showed that all four pulmonary veins enter the left atrium without obstruction. The ASD was not suitable for device closure due to its size and location. A cardiac catheterization was discussed, but the cardiologists refrain from it, since pulmonary vein stenosis had been excluded, device closure was not needed and the PH was regarded to be caused by the left-to-right shunts. A surgical closure of the ASD and PDA was advocated and successfully done at 4 months of age.

A significant rise in the systolic and diastolic arterial blood pressure [mean arterial pressure (MAP) ≥90–105 mm Hg] occurred postoperatively. Cardiac ultrasounds showed sign of a remaining sub-systemic PH after the correction and a decision was made to start sildenafil treatment on the third postoperative day aiming at a target dose of 2 mg/kg/day. Repeated cardiac ultrasounds during the treatment with sildenafil demonstrated a persistent PH at sub-systemic levels evaluated by the tricuspid regurgitant pressure gradient (75 mm Hg, 4 m/s). There was no sign of right ventricular failure. The contractility and the systolic function of the left ventricle were described as good. MAP remained high (> 70–80 mm Hg), when the child was awaken for several weeks after the correction. A diagnosis of severe BPD was confirmed based on chest x-ray (Figure 1) and the definition by the National Institute of Child Health and Human Development program (36, 37). A cystic structure in left lower base was seen on the chest x-ray already the day after birth and it was later defined as a congenital pulmonary adenomatoid malformation (CPAM). Emphysematous changes developed after 35 weeks of postmenstrual age (PMA) (Figure 1). The infant required continuous non-invasive ventilator support using a full face mask and oxygen supplementation with FiO_2_ of 0.5–0.75 to maintain oxygen arterial saturations between 65% and 85%. Despite receiving a sildenafil dose of 1.9 mg/kg/day, the baby developed cyanotic spells with dangerously low to undetectable levels of oxygen saturation and was claimed to have untreatable PH with repeated pulmonary hypertensive crises. It was difficult to increase her feeding due to gastric retentions and what was judge as abdominal spasm or pain. Broad-spectrum antibiotic treatments were given due to the growth of MRSA, and Klebsiella pneumoniae in the upper airways, and rhinovirus was also detected (Table 1).

The infant was considered stable enough to be transferred from the NICU to a general pediatric ward at six months of age. However, her condition deteriorated rapidly after transfer, with severe intercostal respiratory retractions and increased restlessness. Her cyanotic spells became more frequent, despite FiO_2_ of 1.0, and a decision was made to transfer the child to the pediatric intensive care unit (PICU) for optimization of her treatment.

The infant was intubated and underwent tracheostomy, requiring FiO_2_ 1.0 to maintain arterial oxygen saturations of 65%–75%. Despite two weeks of ventilator optimization, pulmonary vasodilators, targeted antibiotics therapy based on culture results, restricted crystalloid infusion, and diuretic treatment, the PH persisted. iNO and iloprost inhalation were administrated as a complement to sildenafil, but only led to transient improvements in oxygenation. Given the lack of response and the opinion that a post-capillary PH was the reason for the clinical deterioration, it was decided to change the treatment strategy from pulmonary vasodilation to systemic afterload reduction. Sildenafil, iNO, and inhaled iloprost were discontinued, and the treatment was switched to the angiotensin II type-1 receptor (AT1R) blocker, losartan. The starting dose was 0.25 mg/kg/dose, increasing to 1 mg/kg/dose twice daily over five days. Anti-inflammatory treatment with melatonin (0.5 mg/kg twice daily) was introduced based on clinical and experimental data supporting its anti-inflammatory properties in BPD (38–40). A low physiological steroid substitution was continued due to iatrogenic adrenal insufficiency, alongside inhaled budesonide (Pulmicort®) (Table 1). Blood volume was optimized with blood products and 5% albumin to stabilized blood pressure alongside the start of losartan, which unmasked a compensated hypovolemia.

Within two to three days after starting losartan, the infant's oxygenation improved, her circulation stabilized, and the repeated cyanotic spells disappeared. Inspired oxygen levels were reduced, sedation was gradually weaned, and enteral feeding was resumed and progressively increased without gastric retention or sign of abdominal pain.

The sign of pulmonary edema on chest x-ray, which occurred during the treatment with pulmonary vasodilators, disappeared within a few days. The discontinuation of the pulmonary vasodilators may partly have attributed to the improvement in oxygenation by decreasing capillary filtration pressure. Her blood pressure normalized and stabilized at a systolic arterial pressure of 60–70 mm Hg (MAP∼50 mm Hg). Cardiac ultrasounds after the alteration of treatment regimen showed normal bilateral ventricular size and function with no remaining direct or indirect indications of pulmonary hypertension.

After 33 days of intensive care, the infant was transferred back to the pediatric ward without clinical or echocardiographic signs of PH. She still required a high-flow nasal-mask during the day and bi-level positive airway pressure at night (FIO_2_ 0.3–0,35) to maintain oxygen saturation >90%. Arrested alveolar development, pruning of vascular bed and cystic changes might contribute to the lasting V/Q mismatch and need of extra oxygen. Over the following year, she gained weight and showed good psychomotor development and no signs of retinopathy. No genetic testing was performed to exclude all possible underlying chromosomal diagnosis contributing to PH, since it was obviously reversible to the treatment.

## 3 Discussion

### 3.1 How RAAS activity influences pro-inflammatory responses and causes lung damage in BPD.

Children with severe BPD and PH often require high oxygen concentrations and mechanical ventilation. Both induce inflammatory responses through oxidative stress and barotrauma (41). Several authors have stressed that this inflammation significantly contributes to the pulmonary damage in BPD (9, 10, 13, 42). Inflammatory responses increase pulmonary and systemic endothelial permeability with subsequent extravascular fluid accumulation and loss of circulatory blood volume (7). Losses of circulatory blood volume in very early born premature infants are also common due to early cord-clamping and the following need of frequent blood sampling. A reduction of intravascular volume decreases systemic cardiac output and serves as an important stimulus for RAAS activation and subsequent increase in systemic vascular resistance. The RAAS cascade is intitated by the cleavage of angiotensinogen to angiotensin I (ANG I) by renin in the kidney. ANG I is subsequently converted to angiotensin II (ANG II) by the enzyme—angiotensin converting enzyme 1 (ACE1) located primarily in pulmonary microvascular endothelium (43). ANG II amplifies several pro-inflammatory responses by activating the angiotensin II type 1 receptor AT1R, leading to immune cell infiltration, augmentation of oxidative stress, increased vascular permeability, and development of pulmonary fibrosis (44, 45). See Table 2 for biological effects of ANG II.

**Table 2:** Pro-inflammatory and hypertensive actions of angiotensin II (ANG II).

Actions by angiotensin II (ANG II)
Disrupting pulmonary endothelial function and increases endothelial permeability (51, 84).
Platelet-leukocyte activation and vascular infiltration of immune cells through the CD40L-CD40 pathway (85).
Induces thrombocytopenia (86).
Amplifies oxidative stress. Increases generation of superoxide annions and hydrogen peroxide (85, 87, 88).
Induces metalloprotease activation, thereby shedding cytokines and receptors from cell surfaces (89).
Induces pulmonary fibrosis (52, 90–92).
Enhances endothelin induced vasoconstriction (93).
Stimulates the release of arginine vasopressin (94).
Physiological concentrations stimulates release of aldosterone (95).
Stimulates synthesis of norepinephrine (96).

The damaging properties of ANG II have been implicated in various lung injuries, including adult respiratory distress syndrome (ARDS), respiratory syncytial virus infection (RSV), and COVID-19 (46–53). ANG II is normally inactivated by angiotensin converting enzyme 2 (ACE2) and a reduction in ACE2 activity has been shown to result in exacerbation of lung injury. Downregulation of ACE2 has been demonstrated during hyperoxia and may be an important contributing factor to inhibit the inactivation of ANG II in BPD (54). Moreover, mitigating the harmful effects of ANG II, by administration of recombinant ACE2, is protective, as is the administration of ARBs, both in clinical studies and in experimental models of lung injury (47, 48, 50, 55).

Elevated levels of ANG II correlates directly with the severity of pulmonary damage and mortality supporting its role in exacerbating lung pathology (51, 53, 56). Increased circulating concentration of ANG II has been found in prematurely born children with very low birth weight (57). Further support för the critical role of ANG II in neonatal pulmonary pathology comes from the observation of the increased incidence of BPD and PH in premature infants exposed to high placental concentrations of ANG II in preeclamptic mothers (58–60). In addition, several reports have noticed an association between early blood losses and the development of BPD and morbidity, which may be explained by the activation of RAAS (61–63). An initial activation of RAAS in extremely premature born children may continue during the following intensive care period due to need of frequent blood sampling, a continuous inflammatory response caused by use of high levels of inspired oxygen, and a high incidence of left-to-right shunts. All these factors contribute to a low systemic cardiac output and consequently RAAS activation, ultimately contributing to lung injury. The activation of ANG II contributes to arterial vasoconstriction and increases afterload of the left ventricle. This may initital cause a subtle left ventricular diastolic dysfunction that can be difficult to detect by the cardiac ultrasound. With time it will increase left atrial pressure, lead to an increased backward pressure in the pulmonary veins and contribute to post-capillary PH (64). PH in infants with BPD usually occurs insidiously and should be followed by repeat cardiac ultrasounds to be detected. In summary, the damage-inducing properties of ANG II aligns well with the key pathophysiological findings in BPD. Data from experimental models and clinical studies provide a strong rationale for using ARBs to block AT1R and thereby potentially mitigate the harmful effects of ANG II in the development of BPD, especially in infants with concurrent PH (34, 54). The amplifying effect of ANG II on oxidative stress, argues for a continuous use of ARB in children with BPD at least until the oxygen demand has been normalized.

### 3.2 How RAAS and its metabolites cause systemic vasoconstriction and pulmonary post-capillary pulmonary hypertension

Angiotensin II is a potent systemic vasoconstrictor resulting in increased arterial blood pressure, which has been observed in children with BPD (30–32, 65, 66). An increase in the afterload of left ventricle increases left ventricular strain, left ventricular end-diastolic pressure, impairs left ventricular diastolic function and elevates left atrial pressure, which in turn is transmitted backward into the pulmonary veins, leading to variable degrees of pulmonary venous congestion (33, 64, 67, 68). This congestive state, albeit not pronounced favors the development of post-capillary PH (68). Elevated left atrial pressure is difficult to diagnose in premature infants given the difficulties to obtain a reliable pulmonary capillary wedge pressure (PCWP) measurement and a significant risk of being underestimated (69). Additionally, typical histological findings in children with BPD confirm that pulmonary venous congestion precedes the diagnosis of PH, indicating that left heart impairment plays a critical role in the pathogenesis of BPD-associated PH (11). The proposed mechanism emphasizes the role of RAAS-mediated systemic vasoconstriction as a key contributor to the pathophysiology of PH in BPD and highlights the potential for targeted therapies directed against AT1R. It may explain why PH in BPD responded promptly to afterload reduction with ARB in the presented case.

### 3.3 The impact of RAAS activation on capillary filtration pressure and the use of pulmonary vasodilators

Pulmonary venous congestion leads to an increase in pulmonary capillary hydrostatic pressure. This raises capillary filtration pressure pushing fluid into the interstitial space of the lung parenchyma. The excess of interstitial fluid increases the diffusion distance for oxygen across the alveolar-capillary membrane, leading to impaired oxygen exchange, oxygen desaturation, and need of oxygen supplementation.

The use of pulmonary vasodilators dilates pulmonary pre-capillary arterioles and exposes the congested pulmonary capillary bed to the pulmonary arterial blood pressure, which increase the capillary filtration pressure further. This may lead to a potential progression of the interstitial fluid leak to a point where the fluid enters the alveolar space. Interstitial lung fluid is difficult to diagnose on a plain chest x-ray, but when the fluid enters the alveolar space it can easily be seen as a pulmonary edema. This was observed in the presented case when the child developed sign of pulmonary edema during the treatment with pulmonary vasodilators. This has led some authors to regard pulmonary vasodilation as contraindicated in BPD (34).

### 3.4 The impact of a limited circulatory blood volume in children with BPD

Clinically, sign of pulmonary edema may potentially lead to the false assessment that the infants are fluid overloaded and need diuresis (70). However, indiscriminate use of diuretics causes a non-compensated loss of intravascular volume and result in further stimulation of the already activated RAAS and, thus a sustained activation of the pathogenic pathway. It may further reduce systemic blood flow, contribute to worsening the pulmonary venous congestion and the development of PH. This scenario explains the importance of maintaining the intravascular volume and circulatory blood volume at the same time as a controlled and restricted crystalloid fluid intake can prevent accumulation of interstitial fluids (71–74).

Infants with BPD and its concurrent inflammatory condition can be characterized to have a compensated hypovolemia, which implies the presence of a reduced circulatory blood volume without obvious signs of hypovolemia. The infants can easily maintain or increase their systemic blood pressure by vasoconstriction at the cost of reduced systemic blood flow. Venoconstriction increases mean systemic fillling pressure, which maintains right atrium pressure and central venous pressure, making these parameters inadequate to be used in the clinical assessment of the circulatory blood volume (75, 76). However, a typical finding in these infants, as in our child, is striking blood pressure fluctuation during sedation and/or feeding, when compensatory protective vasoconstriction is inhibited.

After the introduction of losartan, which potentially inhibits systemic vasoconstriction and thereby mitigates pulmonary venous congestion, the chest x-ray improved immediately and the blood pressure became stable at a normal level for age, simultaneously as the circulatory blood volume was increased with blood products and albumin.

Overall, capillary filtration pressure is a key factor in the respiratory and cardiovascular complications of BPD. Effective management requires careful balancing of fluid intake, circulatory blood volume and pharmacological interventions.

### 3.5 The impact of left to right shunts in BPD

Left-to-right shunts such as PDA, ASD, or patent foramen ovale (PFO) have been noticed in up to 85% of infants with BPD and 97% in severe BPD cases (33). They contribute significantly to the pathophysiology of PH and the development of BPD (77, 78). The shunts allow blood to flow from the systemic to the pulmonary circulation, causing pulmonary overcirculation and systemic hypoperfusion (65). This hypoperfusion is probably the reason why children with left-to-right shunts have very high activity in the RAAS and sympathetic nervous system (79, 80). High activity in RAAS results in the production of ANG II in the lung, which amplify established inflammatory activity (Table 2). Activity in RAAS contributes to arterial vasoconstriction and high sympathetic activity to venoconstriction. Pulmonary overcirculation increases the tone in the pulmonary pre-capillary arterioles by myogenic reflexes, which increases the pulmonary arterial pressure (81, 82). In combination with impaired left ventricular diastolic function and pulmonary venous congestion, it may explain the high incidence of BPD with severe PH in this group of patients (33). It seems, therefore crucial to close left-to-right shunts as soon as possible, especially in children with BPD to normalize systemic blood flow and thereby decrease the activation of RAAS. Normalization of systemic blood flow in a child with a long-standing vasoconstriction and a limited vascular growth will result in a transient high systemic blood pressure, which also was noticed in this case.

Long lasting pulmonary overcirculation remodels pre-capillary arterioles in the lungs to protect against an excessive high capillary filtration pressure. Closing a left-to-right shunt may not immediately normalize pulmonary arterial pressure due to this remodeling. The postoperatively remodeling of smooth muscle in the pulmonary arterioles takes time and requires patience until the pulmonary pressure has normalized. After surgical correction of left-to-right shunt lesions, all children normalize their pulmonary arterial pressure without pulmonary vasodilatory treatment, even if the PH has super-systemic levels postoperative, unless the severe PH was caused by an untreatable severe mitral regurgitation (83). Children with severe PH caused by irreversible lung vascular hypoplasia or a genetic etiology conformable with severe BPD and PH are also irreversible condition without cure by surgical corrections or medical treatments.

Children with PH associated with both a remodeling of the pre-capillary arterioles and underlying pulmonary venous congestion, such as those with BPD, may be particular susceptible to pulmonary vasodilators. Vasodilation of the pulmonary arterioles may easily increase pulmonary capillary filtration pressure and exacerbate the accumulation of interstitial lung fluid. Pulmonary vasodilator treatment was, despite this aspect, started in the NICU in the reported infant, after it was noticed that a significant PH remained postoperatively. This may have contributed to the deterioration in the clinical condition, with the occurrence of interstitial lung fluid and pulmonary edema.

ARB was used instead of an ACE inhibitor, since ARBs directly inhibit the effect of ANG II on the AT1R and therefore have the potential to reduce inflammation, oxidative stress and prevent lung fibrosis in BPD, alongside with its effect to reduce afterload and the pulmonary venous congestion.

In conclusion, our child with BPD and severe PH showed prompt improvement after transitioning the treatment regimen from pulmonary vasodilation to afterload reduction using an ARB. A review of the literature highlights the scientific rationale for employing ARBs in BPD, given their ability to ameliorate pulmonary venous congestion by reducing systemic afterload, lower arterial hypertension, and protect against lung-damaging pro-inflammatory responses mediated by ANG II.

In line with these pathophysiological highlights, it is recommended that ARBs, combined with blood volume optimization, should be considered a first-line treatment for BPD children with PH, particularly in cases associated with high systemic arterial pressure and/or left ventricular diastolic dysfunction. However, despite the pathophysiological benefits favoring the use of ARBs in BPD with PH, the widespread reliance on pulmonary vasodilators in this population underscores the need for a randomized, blinded, controlled trial to compare the efficacy of ARBs against pulmonary vasodilators.

## Data Availability

The data analyzed in this study is subject to the following licenses/restrictions: the data presented in the case was collected from medical records. Requests to access these datasets should be directed to lars.lindberg@med.lu.se.
